# Synergistic influence of education and marriage on the risk for cognition loss among the older people in China

**DOI:** 10.1002/nop2.801

**Published:** 2021-03-16

**Authors:** Ning Sun, Rangcheng Jia, Chunyan Guo, Tongda Sun, Xiaoxin Dong, Long Li, Ping Yang

**Affiliations:** ^1^ NingBo College of Health Sciences Yinzhou China; ^2^ Ningbo City College of Vocation Technology Ningbo Yinzhou China

**Keywords:** education, marriage, older people, senile dementia, severe cognitive impairment

## Abstract

**Aim:**

The study aimed to prove that both rationality and emotion are indispensable for older people to maintain their ability to live independently during the twilight of their lives. The resilience of older people to dementia were investigated by considering the interactions between educational levels and marriage status.

**Design:**

A quantitative study was conducted using questionnaires.

**Methods:**

Four sociodemographic variables (age, sex, educational level and marital status) were collected from 1,177 older Chinese participants, whose mini‐mental state examination scores (MMSE scores) were measured.

**Results:**

A lower educational level coupled with being widowed caused a greater risk for severe cognitive impairment (relative risk [RR] 1.48; 95% confidence interval [CI] 1.20–1.82; *p* < .001) for high‐aged older participants (age range: ≥80) than for their low‐aged counterparts (age range: ≥60 and <80). In contrast, a higher educational level coupled with being married levelled this age‐related risk for cognitive loss (RR 0.91; 95% CI 0.65–1.27; *p* = .62).

## 1 INTRODUCTION

Benefiting from advances in science and technology, humans today have a higher probability of living longer than their predecessors (Partridge et al., 2018). However, in comparison with the acceleration of an ageing population, they also have to face the challenges of increased morbidity, such as the dramatic increase in the number of dementia patients worldwide, which has essentially doubled every 20 years (Ferri et al., 2005; Prince et al., 2013). It is urgent for global researchers to understand the pathological evolution of senile dementia to identify risk factors and develop preventive measures. The active management of senile dementia is necessary to ensure a sustainable future for human societies, since ageing societies are an inevitable demographic trend (Götmark et al., 2018).

## 2 BACKGROUND

Cognitive impairment and dementia make it difficult or impossible for older people to cope with daily activities. According to a hypothesis proposed by Stern (2002), a relatively rich cognitive reserve is critical to understanding resiliency to the progressive evolution of the neuropathology of dementia. The factors contributing to the construction of an adequate cognitive reserve will help delay the onset of senile dementia, whereas a lack of these factors constitutes increasing risks. In addition to genetic factors (Haan et al., 1999), a growing number of modifiable factors have been related to senile dementia. These include occupational complexity (Karp et al., 2009; Qiu et al., 2003; Wajman & Bertolucci, 2010), educational attainment (Allegri et al., 2010; Robitaille et al., 2018; Terrera et al., 2014), marital status (Håkansson et al., 2009), socioeconomic status (Osler et al., 2013) and social networks (Bassuk et al., 1999; Fratiglioni et al., 2004; Kuiper et al., 2015; Saczynski et al., 2006).

Just recently, a data analysis report integrating six longitudinal studies demonstrated that older individuals with high educational attainment and high socioeconomic statuses have remarkably longer non‐cognitively impaired life expectancies as opposed to those with low educational attainment and low socioeconomic statuses (Robitaille et al., 2018). This observation suggests that education and socioeconomic status are synergistic resilience factors against the onset of senile dementia. Based on these implications, we sought to further explore the possibility of a synergistic effect on the risk for cognition loss through education and another resilience factor––marriage––among older people. Compared to those possessing a higher socioeconomic status, it is considered normative for ordinary people to sustain a marriage––which is regarded as one of the strongest interpersonal relationships in an individual's social network. Education can enable people to be more rational and make higher‐quality decisions in their lives (Kim et al., 2018). However, we should not ignore the strong emotional needs of older people (van der Steen et al., 2018). In this study, we aim to prove that both rationality and emotion are indispensable for older people to maintain their ability to live independently during the twilight of their lives.

## 3 METHODS

### 3.1 Study design

This quantitative study was conducted using questionnaires.

### 3.2 Setting and sample

From July 2018–September 2018, our research team visited five 5‐level professional pension institutions that were located in Ningbo city to recruit 1,300 older people aged 60 years or older by convenience sampling. “5‐level” implies that these facilities meet the highest level of care facility standards in China. The following inclusion criteria were used: (a) from local households and older than 60 years of age; (b) met the Diagnostic Criteria for Dementia (4th edition of the *American Diagnostic and Statistical Manual of Mental Disorders*). Mild cognitive impairment or severe cognitive impairment (SCI) was diagnosed in the Li Huili Hospital of Ningbo Medical Center.

The questionnaire was returned by 1,250 patients (94.2% response rate). 73 questionnaires were left blank and were therefore excluded. The final number of analysed questionnaires was 1,177.

### 3.3 Data collection

Ten investigators underwent unified training. The data collection was conducted by face‐to‐face interviews. All the older adults with dementia were included in this study after a scheduled meeting arranged by nursing home and community service centre managers according the list of clinical diagnoses. The investigators explained the research objectives and methods and obtained written consent and cooperation from older adults with dementia and their families who met the inclusion and exclusion criteria. The older adults with dementia who consented to participate received an envelope containing a packet with the questionnaires. Participants completed the questionnaires immediately upon receipt and replaced them in the envelope for collection by the investigators. To ensure anonymity, code numbers were placed on the completed questionnaires after their return to the investigators.

### 3.4 Study measures

For each participant, four sociodemographic variables (age, sex, educational level and marital status) were collected by the researchers and verified by checking the electronic medical records of their respective professional pension institutions. All participants were divided into two groups by age: the low‐aged older people group (age range: ≥60 and <80) and the high‐aged older people group (age range: ≥80). Participants who were illiterate or had completed only elementary or junior high school education were classified as having low educational levels. Those who had completed high school or some college education were labelled as having a high educational level. Five distinct marital statuses of the patients were recorded. These included single, widowed, divorced, married and cohabitating with others. Accompanied by a trained researcher, each participant was required to complete the mini‐mental state examination (MMSE) (Folstein et al., 1975).

### 3.5 Ethical statement

The ethics committee of the College (NBWY‐010) approved this study. Prior to enrolment in the study, all participants or their guardians were informed of the research plan and signed a written statement of informed consent. The only prerequisite for a guardian to sign the informed consent statement on behalf of a participant was that the participant could not sign the consent form independently due to an impaired cognitive function.

### 3.6 Data analysis

All data are expressed using the mean ± *SD* (Standard Deviation). The software Graphpad Prism version 6.0 (GraphPad Software, Inc., La Jolla, CA, USA) was used to perform Fisher's exact test, followed by a calculation of the relative risk (RR) of SCI, a Student's *t* test and a one‐way analysis of variance (ANOVA) test. The abbreviation RR refers to the relative risk of being SCI in the high‐aged group compared with that in the low‐aged group. Differences were only considered to be significant at *p* < .05.

## 4 RESULTS

### 4.1 The percentage of widowhood was significantly high among high‐aged older people

We collected sociodemographic information from a total of 1,177 older Chinese people that were living in professional pension institutions and evaluated their cognitive functioning levels using the MMSE test. More than 60% of the older people were 80 years or older (Table 1). The MMSE‐evaluated cognitive functioning of the low‐aged older people was noticeably better than that of the high‐aged counterparts (*p* < .001). There was no difference in the proportion of people with high education between the two groups. No participant reported their marital status as “single,” “divorced” or “cohabitating with others.” In the low‐aged older people group, about 57% of the participants were widowed. Comparably, seven of ten high‐aged older people were widowed. A significant difference between the two groups was noted in this percentage (*p* < .001). We did not find a gender‐specific difference in the percentage of widowed people within the high‐aged older people group. The percentage of widowed men was 70.7%, and the percentage of widowed women was 71.8%.

**TABLE 1:** Participant characteristics

Characteristics	Low age (*N* = 432)	High age (*N* = 745)
Age (years) mean (*SD*)	71.5 (5.6)	86.9 (4.7)
Sex (male/female, %)	51.6%/48.4%	37.3%/62.7%*
Educational level (low/high, %)	68.8%/31.2%	71.4%/28.6%*
Marital status (widowed/married, %)	56.9%/43.1%	71.0%/29.0%
MMSE score (mean, *SD*)	7.8 (9.0)	5.1 (7.3)^#^

Note

Low age: the low‐aged older people group (age range: ≥60 and <80), High age: the high‐aged older people group (age range: ≥80).

* Fisher's exact test *p* < .001, versus Low‐age older people group.

^#^ Student's *t* test *p* < .001, versus Low‐age older people group.

### 4.2 Higher educational level and being married decreased the age‐related risk for severe cognitive impairment

We evaluated the influence of education and marriage on the incidence of severe cognitive impairment. Widowhood rather than being married significantly increased the age‐related risk for severe cognitive impairment (RR 1.46; 95% confidence interval [CI] 1.23–1.74; *p* < .001, Table 2). Compared with well‐educated older people, poorly educated older people had a higher age‐related risk for severe cognitive impairment (RR 1.36; 95% CI 1.17–1.59; *p* < .001, Table 3). When education and marriage were assessed jointly, the age‐related risk for severe cognitive impairment was completely offset by a higher educational level coupled with being married (RR 0.91; 95% CI 0.65–1.27; *p* = .67, Table 4). However, poorly educated and widowed older people had the highest age‐related risk for severe cognitive impairment (RR 1.48; 95% CI 1.20–1.82; *p* < .001, Table 4).

**TABLE 2:** Influence of marriage on the incidence of severe cognitive impairment

Marital status	Low age (SCI/non‐SCI)	High age (SCI/non‐SCI)	RR (95% CI)	*p* value
Widowed	95/151	298/231	1.46 (1.23–1.74)	<.001
Married	89/97	115/101	1.11 (0.92–1.35)	.32

Abbreviation: SCI, severe cognitive impairment.

**TABLE 3:** Influence of education on the incidence of severe cognitive impairment

Educational level	Low age (SCI/non‐SCI)	High age (SCI/non‐SCI)	RR (95% CI)	*p* value
Low	122/175	298/234	1.36 (1.17–1.59)	<.001
High	62/73	115/101	1.18 (0.94–1.47)	.15

Abbreviation: SCI, severe cognitive impairment.

**TABLE 4:** Joint influence of marriage and education on the incidence of severe cognitive impairment

Marital status/Educational level	Low age (SCI/non‐SCI)	High age (SCI/non‐SCI)	RR (95% CI)	*p* value
Widowed/Low	67/111	218/174	1.48 (1.20–1.82)	<.001
Married/High	34/33	35/41	0.91 (0.65–1.27)	.62

Abbreviation: SCI, severe cognitive impairment.

### 4.3 Well‐educated and married high‐aged older people appeared resilient to senile dementia

**FIGURE 1:**
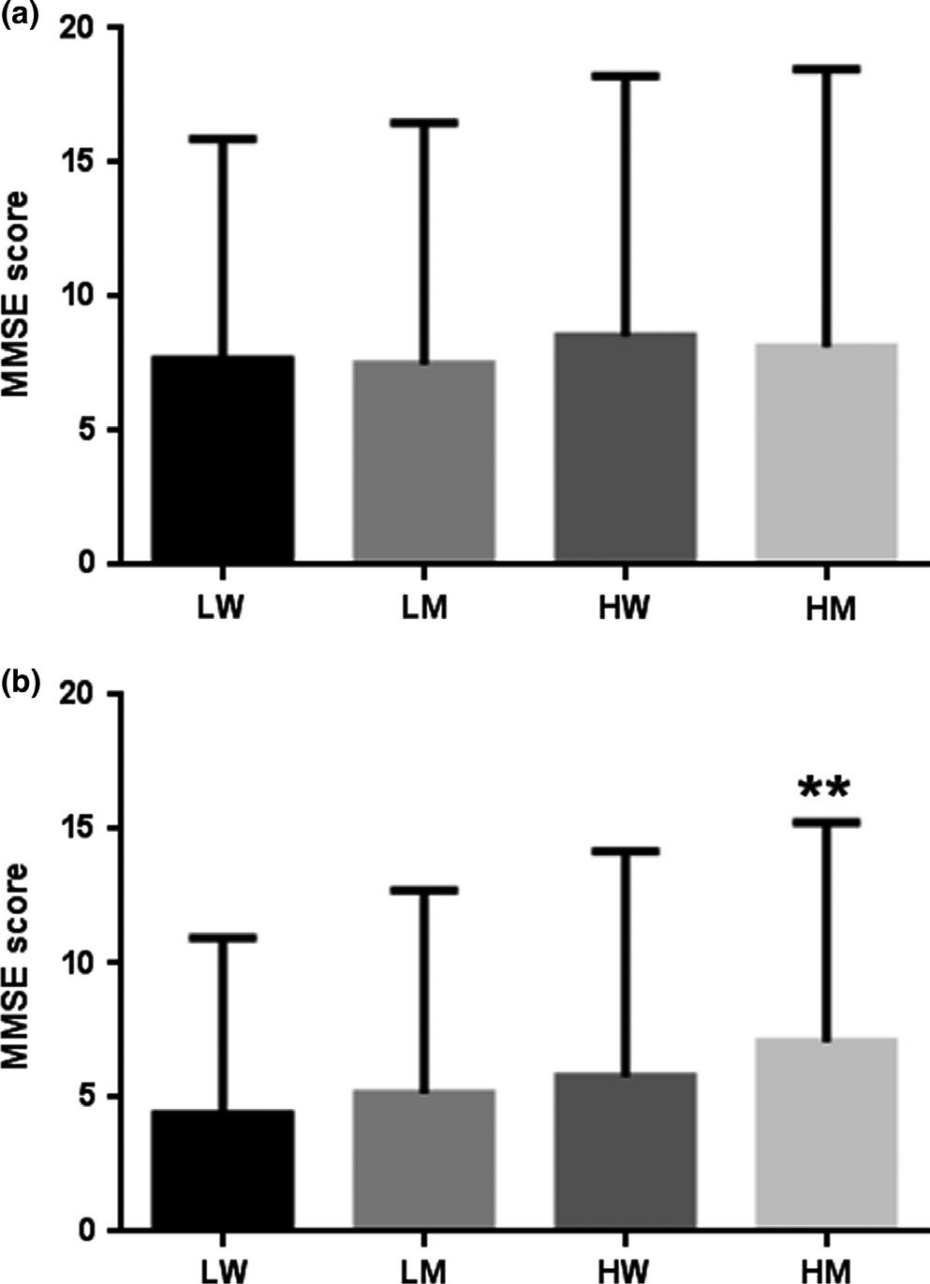
The MMSE score among the four subgroups of low‐aged older people

To assess the cognitive impact of education and marriage, participants were further divided into four subgroups: low educational level and widowed (LW), low educational level and married (LM), high educational level and widowed (HW) and high educational level and married (HM). No significant difference was observed in the MMSE score among the four subgroups of low‐aged older people (Figure 1a). Comparably, the mean MMSE score of HM older people was obviously higher than that of the LW counterparts (*p* < .01, Figure 1b). Further comparisons between the corresponding subgroups in the two age groups revealed obvious age‐related cognitive decline in LW, LM and HW older people compared to HM older people.

## 5 DISCUSSION

By dividing the participants recruited for this study into two groups, significant differences were observed in the proportion of widowed population rather than in the proportion of those with high levels of education. Consistent with previous studies in the United States and South Korea (Bae et al., 2015; Steenland et al., 2009), our findings suggest a higher risk for severe cognitive impairment among unmarried older people as opposed to those who are married. In our study, only one of the three most common unmarried statuses (single, divorced and widowed) was investigated since our survey indicated that single and divorced older people were rare compared to those who were widowed. Using the incidence rates for mild cognitive impairment among older married individuals as a baseline, Brenowitz et al. (2014) found that only the widowed––rather than the single or divorced––held a significant risk for mild cognitive impairment. Therefore, it is reasonable to suggest that widowed older people should be investigated separately rather than in conjunction with those who are single or divorced. Despite women having longer life expectancies than men (Fei et al., 2017), we found that high‐aged older people men and women had similar high widowhood rates. This finding suggests that it is more difficult to still continue marriage at an advanced age than to extend the life of high‐aged older people individuals.

For older people with mild cognitive impairment, living in a professional pension institution is not as cost‐effective as living at home (Kraft et al., 2010). However, we learned through dialogue that avoiding becoming a burden to their children, rather than economic efficiency, was the primary reason governing older people's decision to choose institutional care. Concern over cognitive decline is the leading reason behind older people choosing institutional care (Werner & Segel‐Karpas, 2016). Our observations indicate that the cognitive functioning of the older people living in professional pension institutions was generally poor. This finding is consistent with the assertions of Werner and Segel‐Karpas (2016), who noted that signs of dementia caused older people to worry about dementia and adopt this uneconomical behaviour. Other possible explanations for this are that a residential shift from familiar surroundings to an unfamiliar place might have a negative impact on cognitive functioning (Fong et al., 2012). Subsequently, the weak interpersonal relationships established in institutions can hardly replace the strong emotional support that comes from marriage. The substitution of a strong interpersonal relationship with multiple weak interpersonal relationships has been demonstrated as being harmful to cognitive functioning (Brenowitz et al., 2014). Nevertheless, our results suggest that maintaining one's married status in old age is beneficial to maintaining cognition in comparison with being widowed, even if there are deficiencies in professional pension institutions. Results of a recent study show that cognitive decline is not inevitable among at‐risk older people persons if continuous lifestyle interventions can be adopted (Kulmala et al., 2019). Compared to other high‐aged people, married high‐aged participants with high levels of education were identified as having a lower risk for severe cognitive impairment. This finding was consistent with previous studies (Larsson et al., 2017; Wolf et al., 2019). Segmentation of the participants implies that married low‐aged older people with high levels of education have the lowest risk of suffering from severe cognitive impairment when they are over 80 years old. Our results suggest that in this subpopulation, the demographical indicators of education and marriage demonstrate a synergistic prevention pattern against severe cognitive impairment. However, it should be noted that this lower risk was only the result of their comparison with the high‐aged group. Compared to other people in the low‐aged group, these people did not show a lower rate of severe cognitive impairment.

### 5.1 Limitations and further research

There are some limitations to this study. First, the sample size of this study is small. It only focused on the older people living in professional pension institutions and did not investigate the older people living at home. Therefore, the main conclusion identifying an age‐related education‐marriage synergetic relationship should be considered with caution and requires further validation by larger‐scale panel studies. Second, clinical diagnoses of severe cognitive impairment were not used in the study. Relying solely on the MMSE measures may possibly result in the misclassification of research participants. Third, because this is a cross‐sectional study, it is difficult to determine the exact role of education‐marriage synergy in delaying cognitive decline in older people from the perspective of individualization. Fourth, additional factors related to cognitive decline, including cardiovascular and cerebrovascular diseases, tobacco and alcohol use, and lifestyle, were not evaluated or used for data segmentation in this study.

## 6 CONCLUSION

This cross‐sectional study reveals that education alone does not sufficiently address the complexity of the risk factors against age‐related cognitive decline. Maintaining the integrity of marriage is also necessary for ensuring behavioural independence in the twilight of older life. This age‐related synergy between education and marriage against cognitive decline suggests that a stable and strong interpersonal relationship is important for the growing subpopulation of long‐lived older people with high levels of education. The benefits of marriage become more apparent as people age. Further comparative studies are needed to investigate the potential impact of this trend on senile dementia.

## CONFLICT OF INTEREST

The authors declare that they have no involvement, financial or otherwise that may potentially bias their work.

## AUTHOR CONTRIBUTIONS

The authors were responsible for the paper as follows: NS: Conception, design, analysis, and data interpretation, drafting the manuscript, revising the manuscript and its final approval. LL: Acquisition of data, project administration, manuscript revisions and its final approval. CYG and PY: Formal analysis, manuscript revision and final approval. TDS and XXD: Conception, manuscript revision and final approval. RCJ: Conception, design, funding acquisition, project administration, manuscript revision and final approval. All the authors have read and approved the final manuscript.

## Data Availability

The data sets generated and analysed during the current study are not publicly available due to ethical restrictions and patient confidentiality but are available from the corresponding author on reasonable request. The aggregated data are provided in the tables.

## References

[nop2801-bib-0001] Allegri, R. F., Taragano, F. E., Krupitzki, H., Serrano, C. M., Dillon, C., Sarasola, D., Feldman, M., Tufró, G., Martelli, M., & Sanchez, V. (2010). Role of cognitive reserve in progression from mild cognitive impairment to dementia. Dementia & Neuropsychologia, 4, 28–34. 10.1590/S1980-57642010DN40100005 29213657 PMC5619527

[nop2801-bib-0002] Bae, J. B., Kim, Y. J., Han, J. W., Kim, T. H., Park, J. H., Lee, S. B., Lee, J. J., Jeong, H. G., Kim, J. L., Jhoo, J. H., Yoon, J. C., & Kim, K. W. (2015). Incidence of and risk factors for Alzheimer's disease and mild cognitive impairment in Korean older. Dementia and Geriatric Cognitive Disorders, 39, 105–115. 10.1159/000366555 25401488

[nop2801-bib-0003] Bassuk, S. S., Glass, T. A., & Berkman, L. F. (1999). Social disengagement and incident cognitive decline in community‐dwelling older persons. Annals of Internal Medicine, 131, 165–173. 10428732 10.7326/0003-4819-131-3-199908030-00002

[nop2801-bib-0004] Brenowitz, W. D., Kukull, W. A., Beresford, S. A., Monsell, S. E., & Williams, E. C. (2014). Social relationships and risk of incident mild cognitive impairment in U.S. Alzheimer's disease centers. Alzheimer Disease and Associated Disorders, 28, 253–260. 10.1097/WAD.0000000000000020 24577205 PMC4139444

[nop2801-bib-0005] Fei, F. R., Zhong, J. M., Yu, M., Gong, W. W., Wang, M., Pan, J., Wu, H. B., & Hu, R. Y. (2017). Impact of injury‐related mortality on life expectancy in Zhejiang, China based on death and population surveillance data. BMC Public Health, 18, 24. 10.1186/s12889-017-4566-3 28716017 PMC5513166

[nop2801-bib-0006] Ferri, C. P., Prince, M., Brayne, C., Brodaty, H., Fratiglioni, L., Ganguli, M., Hall, K., Hasegawa, K., Hendrie, H., Huang, Y., Jorm, A., Mathers, C., Menezes, P. R., Rimmer, E., Scazufca, M., & Alzheimer's Disease International (2005). Global prevalence of dementia: A Delphi consensus study. Lancet, 366, 2112–2117. 10.1016/S0140-6736(05)67889-0 16360788 PMC2850264

[nop2801-bib-0007] Folstein, M. F., Folstein, S. E., & McHugh, P. R. (1975). “Mini‐mental state”. A practical method for grading the cognitive state of patients for the clinician. Journal of Psychiatric Research, 12, 189–198. 1202204 10.1016/0022-3956(75)90026-6

[nop2801-bib-0008] Fong, T. G., Jones, R. N., Marcantonio, E. R., Tommet, D., Gross, A. L., Habtemariam, D., Schmitt, E., Yap, L., & Inouye, S. K. (2012). Adverse outcomes after hospitalization and delirium in persons with Alzheimer disease. Annals of Internal Medicine, 156, 848–856. 10.7326/0003-4819-156-12-201206190-00005 22711077 PMC3556489

[nop2801-bib-0009] Fratiglioni, L., Paillard‐Borg, S., & Winblad, B. (2004). An active and socially integrated lifestyle in late life might protect against dementia. Lancet Neurology, 3, 343–353. 10.1016/S1474-4422(04)00767-7 15157849

[nop2801-bib-0010] Götmark, F., Cafaro, P., & O'Sullivan, J. (2018). Aging human populations: Good for us, good for the earth. Trends in Ecology & Evolution, 33, 851–862. 10.1016/j.tree.2018.08.015 30340868

[nop2801-bib-0011] Haan, M. N., Shemanski, L., Jagust, W. J., Manolio, T. A., & Kuller, L. (1999). The role of APOE epsilon4 in modulating effects of other risk factors for cognitive decline in older persons. JAMA, 282, 40–46. 10.1001/jama.282.1.40 10404910

[nop2801-bib-0012] Håkansson, K., Rovio, S., Helkala, E. L., Vilska, A. R., Winblad, B., Soininen, H., Nissinen, A., Mohammed, A. H., & Kivipelto, M. (2009). Association between mid‐life marital status and cognitive function in later life: Population based cohort study. British Medical Journal, 339, b2462. 10.1136/bmj.b2462 19574312 PMC2714683

[nop2801-bib-0013] Karp, A., Andel, R., Parker, M. G., Wang, H. X., Winblad, B., & Fratiglioni, L. (2009). Mentally stimulating activities at work during midlife and dementia risk after age 75: Follow‐up study from the Kungsholmen Project. American Journal of Geriatric Psychiatry, 17, 227–236. 10.1097/JGP.0b013e318190b691 19454849

[nop2801-bib-0014] Kim, H. B., Choi, S., Kim, B., & Pop‐Eleches, C. (2018). The role of education interventions in improving economic rationality. Science, 362, 83–86. 10.1126/science.aar6987 30287661

[nop2801-bib-0015] Kraft, E., Marti, M., Werner, S., & Sommer, H. (2010). Cost of dementia in Switzerland. Swiss Medical Weekly, 140, w13093. 10.4414/smw.2010.13093 22250014

[nop2801-bib-0016] Kuiper, J. S., Zuidersma, M., Oude Voshaar, R. C., Zuidema, S. U., van den Heuvel, E. R., Stolk, R. P., & Smidt, N. (2015). Social relationships and risk of dementia: A systematic review and meta‐analysis of longitudinal cohort studies. Ageing Research Reviews, 22, 39–57. 10.1016/j.arr.2015.04.006 25956016

[nop2801-bib-0017] Kulmala, J., Ngandu, T., Havulinna, S., Levälahti, E., Lehtisalo, J., Solomon, A., Antikainen, R., Laatikainen, T., Pippola, P., Peltonen, M., Rauramaa, R., Soininen, H., Strandberg, T., Tuomilehto, J., & Kivipelto, M. (2019). The effect of multidomain lifestyle intervention on daily functioning in older people. Journal of the American Geriatrics Society, 67, 1138–1144. 10.1111/jgs.15837 30809801

[nop2801-bib-0018] Larsson, S. C., Traylor, M., Malik, R., Dichgans, M., Burgess, S., Markus, H. S., CoSTREAM Consortium , & on behalf of the International Genomics of Alzheimer's Project (2017). Modifiable pathways in Alzheimer's disease: Mendelian randomisation analysis. British Medical Journal, 359, j5375. 10.1136/bmj.j5375 29212772 PMC5717765

[nop2801-bib-0019] Osler, M., Avlund, K., & Mortensen, E. L. (2013). Socio‐economic position early in life, cognitive development and cognitive change from young adulthood to middle age. European Journal of Public Health, 23, 974–980. 10.1093/eurpub/cks140 23093718

[nop2801-bib-0020] Partridge, L., Deelen, J., & Slagboom, P. E. (2018). Facing up to the global challenges of ageing. Nature, 561, 45–56. 10.1038/s41586-018-0457-8 30185958

[nop2801-bib-0021] Prince, M., Bryce, R., Albanese, E., Wimo, A., Ribeiro, W., & Ferri, C. P. (2013). The global prevalence of dementia: A systematic review and metaanalysis. Alzheimers & Dementia, 9, 63–75.e2. 10.1016/j.jalz.2012.11.007 23305823

[nop2801-bib-0022] Qiu, C., Karp, A., von Strauss, E., Winblad, B., Fratiglioni, L., & Bellander, T. (2003). Lifetime principal occupation and risk of Alzheimer's disease in the Kungsholmen project. American Journal of Industrial Medicine, 43, 204–211. 10.1002/ajim.10159 12541276

[nop2801-bib-0023] Robitaille, A., van den Hout, A., Machado, R. J. M., Bennett, D. A., Čukić, I., Deary, I. J., Hofer, S. M., Hoogendijk, E. O., Huisman, M., Johansson, B., Koval, A. V., van der Noordt, M., Piccinin, A. M., Rijnhart, J. J. M., Singh‐Manoux, A., Skoog, J., Skoog, I., Starr, J., Vermunt, L., … Muniz, T. G. (2018). Transitions across cognitive states and death among older adults in relation to education: A multistate survival model using data from six longitudinal studies. Alzheimers & Dementia, 14, 462–472. 10.1016/j.jalz.2017.10.003 PMC6377940 29396108

[nop2801-bib-0024] Saczynski, J. S., Pfeifer, L. A., Masaki, K., Korf, E. S., Laurin, D., White, L., & Launer, L. J. (2006). The effect of social engagement on incident dementia: The Honolulu‐Asia Aging Study. American Journal of Epidemiology, 163, 433–440. 10.1093/aje/kwj061 16410348

[nop2801-bib-0025] Steenland, K., MacNeil, J., Vega, I., & Levey, A. (2009). Recent trends in Alzheimer disease mortality in the United States, 1999 to 2004. Alzheimer Disease & Associated Disorders, 23, 165–170. 10.1097/WAD.0b013e3181902c3e 19484918 PMC2719973

[nop2801-bib-0026] Stern, Y. (2002). What is cognitive reserve? Theory and research application of the reserve concept. Journal of the International Neuropsychological Association, 8, 448–460. 10.1017/S1355617702813248 11939702

[nop2801-bib-0027] Terrera, G. M., Minett, T., Brayne, C., & Matthews, F. E. (2014). Education associated with a delayed onset of terminal decline. Age and Ageing, 43, 26–31. 10.1093/ageing/aft150 24136340 PMC3861340

[nop2801-bib-0028] van der Steen, J. T., Smaling, H. J., van der Wouden, J. C., Bruinsma, M. S., Scholten, R. J., & Vink, A. C. (2018). Music‐based therapeutic interventions for people with dementia. Cochrane Database of Systematic Reviews, 7, CD003477. 10.1002/14651858.CD003477.pub4 PMC6513122 30033623

[nop2801-bib-0029] Wajman, J. R., & Bertolucci, P. H. F. F. (2010). Intelectual demand and formal education as cognitive protection factors in Alzheimer's disease. Dementia & Neuropsychologia, 4, 320–324. 10.1590/S1980-57642010DN40400011 29213705 PMC5619066

[nop2801-bib-0030] Werner, P., & Segel‐Karpas, D. (2016). Factors associated with preferences for institutionalized care in older persons: Comparing hypothetical conditions of permanent disability and Alzheimer's disease. Journal of Applied Gerontology, 35, 444–464. 10.1177/0733464814546041 25245385

[nop2801-bib-0031] Wolf, D., Fischer, F. U., Fellgiebel, A., & Alzheimer's Disease Neuroimaging Initiative (2019). A methodological approach to studying resilience mechanisms: Demonstration of utility in age and Alzheimer's disease‐related brain pathology. Brain Imaging and Behavior, 13, 162–171. 10.1007/s11682-018-9870-8 29713998

